# Cancer of the Cervix

**Published:** 1888-04

**Authors:** 


Cancer of the Cervix.—Clinically, three cases can be distinguished: (1) The existence of an infiltration, diffuse, or limited to the cervix, without ulceration. One would willingly believe it a sclerous metritis or a myoma of the cervix. Absence of ulceration and the even distribution of the induration plead against cancer, but the actual truth cannot be obtained but by a microscopic examination of a piece of the tissue. (2) The cervix is the seat of an ulceration accessible to the finger, and to the eye by means of a speculum. If ulceration rest upon an indurated base, and if the borders are equally indurated, the diagnosis of cancer is undoubted. It is the induration which above all constitutes the chief element in diagnosis. Ulceration can assume other very different aspects. (3) Infiltration and ulceration occupying the most elevated portion of the cervix, whose visible parts appear bloody. Here cancer can be only suspected. The diagnosis can only be established in a positive manner by dilation of the uterine orifice after the method of Vulliet. To the diagnosis of cancer itself must be added that of the kind and extent of the affection. The kind is of slight practical interest, as it consists of an epithelioma or carcinoma, and the prognosis does not differ in the two cases. It is different, however, with the extent of the disease. Touch, which reveals the local condition, the mobility of the uterus, and lastly, a study of the phenomena of the pain, lead us to this diagnosis.—*L' Union Medical—Quar. Comp.*

